# YKL-40 regulated epithelial-mesenchymal transition and migration/invasion enhancement in non-small cell lung cancer

**DOI:** 10.1186/s12885-015-1592-3

**Published:** 2015-08-15

**Authors:** Malvin Jefri, Yi-Ning Huang, Wen-Chien Huang, Chun-San Tai, Wen-Liang Chen

**Affiliations:** 1 Department of Biological Science and Technology, National Chiao Tung University, Hsinchu, 1001 University Road, Hsinchu, Taiwan 300 ROC; 2 Institute of Bioinformatics and Systems Biology, National Chiao Tung University, Hsinchu, Taiwan; 3 Institute of Traditional Medicine, National Yang Ming University, Taipei, Taiwan; 4 Department of Thoracic Surgery, Mackay Memorial Hospital, Taipei, Taiwan

## Abstract

**Background:**

YKL-40 is a secreted inflammatory protein that its overexpression has been reported to correlate with poor outcome of various malignant diseases, especially in cancer. However, the function of this protein is still unclear.

**Methods:**

The clinical prognosis of non-small cell lung cancers (NSCLC) patients and their clinical YKL-40 expressions were obtained from the Prognoscan database. The expressions of YKL-40 in patient samples were determined by Western Blotting assay. YKL-40 gene knockdown and overexpression were performed on NSCLC cancer cells (CL1-1 and CL1-5). The cells were investigated for their epithelial–mesenchymal transition (EMT) markers gene modulation through Western Blotting and RT-PCR. Further cell metastatic abilities were assessed by transwell migration and invasion assay.

**Result:**

In this study, YKL-40 was observed to be highly expressed in NSCLC specimens. Furthermore, determined by the PrognoScan database analysis, patients with high expression levels of YKL-40 were found with poor prognosis. In the in vitro study, different characteristics of NSCLC cell lines (CL1-1, H23, H838, CL1-5, and H2009) were used as study models, where YKL-40 expression levels were determined to correlate with the phenotypic characteristics of cancer metastasis. In this study,YKL-40 was demonstrated to regulate EMT marker expressions such as Twist, Snail, Slug, N-cadherin, Vimentin, and E-cadherin. The protein’s affects in cancer cell migration and invasion were also observed in YKL-40 overexpression or knock down NSCLC cell lines.

**Conclusion:**

All of results from this study suggest that YKL-40 is a major factor in NSCLC metastasis. Thus, YKL-40 may serve as therapeutic targets for NSCLC patients in the future.

## Background

Lung cancer is the leading cause of cancer death and its prevention is a major worldwide challenge. Non-small cell lung cancer (NSCLC) accounts for more than 80 % of lung cancers [1, 2]. The classifications of NSCLC include large cell carcinoma, adenocarcinoma, and squamous cell carcinoma. Approximately one-half of lung cancers become metastatic, and the most common sites of metastasis are found in the lymph nodes, liver, adrenal glands, bone, and brain. The most frequently reported lung carcinoma subtype is lung adenocarcinoma. In the past decades, many genes have been found to be modulated in cancer cells. These proteins expressed from these modulated genes, such as Wnt5A, KFL6, cyclin D1, ER-α, UPA, PAI-1, HER2, and c-myc, were determined with essential roles in cancer initiation and progression [3–5]. Among the upregulated proteins, YKL-40 was frequently proposed for its role in cancer metastasis [6–8].

YKL-40, a member of mammalian chitinase-like proteins, is overexpressed in many pathological conditions that includes fibroblastic change in liver cirrhosis, increased deposition of connective tissue components and hyperplastic synovium in rheumatoid arthritis, and increased cellular infiltration as well as epithelial proliferation in chronic colitis [4]. YKL-40 is also up-regulated in many chronic inflammatory conditions such as inflamed tissues in ulcerative colitis, Crohn’s disease, rheumatoid arthritis, osteoarthritis, asthma, chronic obstructive pulmonary disease (COPD), and liver cirrhosis. The levels of YKL-40 were determined to be upregulated in in solid cancers arising from bone, brain, breast, cartilage, cervix, colon, germ cell, head and neck, kidney, liver, lung, lymph node, ovary, pancreas, prostate, skin, and thyroid, when compared with their respective normal tissues or cells [5–7]. Aside from its association with inflamed tissues, YKL-40 expression is also regulated by many inflammatory cytokines. It is believed that inflammatory mediators such as pro-inflammatory cytokines of IL-1, TNF-α, IL-6, and IL-13, regulate YKL-40 expression in inflammatory conditions [8]. Furthermore, YKL-40 expression is also controlled by hormones such as vasopressin, and parathyroid hormone-related protein in both macrophages and epithelial cells [9, 10]. Despite the association of increased expression of YKL-40 with many diseases, its biological function is still largely unknown. Further studies have determined that YKL-40 interacts with many components of the extracellular matrix (ECM), including hyaluronic acid, fibronectin, and collagen I, II, III and IV [11]. The ability to adhere to extracellular matrix is especially important for cancer cell migration and invasion [4, 12]. YKL-40 protein is involved in proliferation of fibroblasts and modulation of collagen formation, facilitating tumor invasion, and metastasis [13]. These mesenchymal functions have been verified in a microarray analysis with high-grade gliomas. The analyses determined that YKL-40 is upregulated, together with other genes, in mesenchymal tissues and are associated with poor prognosis (adult malignant gliomas) [14, 15]. However, the function of YKL-40 in cancer metastasis is still unclear.

Therefore, this study, was aimed to investigate the relationship between YKL-40 and tumor migration and invasion in NCLC. The phenotypic characteristics of YKL-40 NSCLC cell lines were determined and compared. Further analysis was performed to determine the influence of YKL-40 in regulating EMT through the AKT signaling pathways for enhanced cancer migration and invasion. We believe that YKL-40 may serve as therapeutic targets for NSCLC patients in the future.

## Materials and methods

### Patients and tissue samples

Ten de-identified tumor samples (each includes NSCLC and normal control patients) were obtained from the Shuang Ho Hospital Resource Center (New Taipei city, Taiwan). The patients in this study were free from any inflammatory conditions. Written informed consents were obtained from the patients and this study was approved by the ethics committee of Shuang Ho Hospital.

### NSCLC cell lines and cell culture

Human adenocarcinoma cell lines H838, H23, and H2009 were purchased from American Type Culture Collection (Manassas, VA), while CL1-1 and CL 1–5 were kind gifts from Dr. Pan-Chyr Yang of National Taiwan University. Cells were cultured in RPMI 1640 medium (GIBCO, Gaithersburg, MD) containing 10 % fetal bovine serum (FBS) (Jacques Boy, Reims, France), 100 U/mL of penicillin (GIBCO) and 100 U/mL of streptomycin (GIBCO) at 37 °C in a 5 % CO2 atmosphere at 99 % humidity.

### Generation of YKL-40 stable expression CL1-1 cell slines

The full length of YKL-40 cDNA was isolated directly from a NSCLC CL1-5 cDNA library using the polymerase chain reaction (PCR) and introduced restriction sites for BamHI and HindIII. After sequence confirmation, the full-length YKL-40 was cloned into the reporter vector pEGFP-C1 to yield the reporter plasmid. The NSCLC CL1-1 cells were transfected with 2 μg of YKL-40 or vector control DNA using TurboFect™ reagent (Fermentas, Glen Burnie, MD) as the delivery vehicle. Selection with 800 μg/ml of G418 was performed at 24 h after transfection, and the drug-resistant cell populations were used for subsequent studies.

### Generation of YKL-40 stable knockdown CL1-5 cell lines

TurboFect™ (Fermentas, Glen Burnie, MD) was used to deliver plasmid DNA containing small hairpin RNA (shRNA) against human YKL-40(OriGene, Rockville, MD) into NSCLC CL1-5 cells in a 24-well culture plate. Selection with puromycin was performed at 24 h after transfection, and the drug-resistant cell populations were used for subsequent studies.

### Generation of YKL-40 stable knockdown and re-overexpression CL1-5 cell lines

After the stable culture YKL-40 knockdown CL1-5 cells, the cells were transfected with 2 μg reporter vector (DsRed plasmid), which contained the full-length YKL-40 gene, using TurboFect™ reagent (Fermentas, Glen Burnie, MD) as the delivery vehicle. Selection with 800 μg/ml of G418 was performed at 24 h after transfection, and the drug-resistant cell populations were used for subsequent studies.

### Migration assay

Cells were harvested and suspended in RPMI 1640 medium containing 10 % FBS at a concentration of 1 × 10^6^ cells/mL. A transwell apparatus with 8 μm pore size membrane (Millipore, Billerica, MA) was used to analyze the migration activity. In brief, suspension of cells in 100 μL of serum-free RPMI 1640 medium were seeded into the upper chamber of the apparatus, 250 μL of RPMI containing 10 % FBS were added to the insert well, and the apparatus was incubated at 37 °C for 6 h. After incubation, the inner wall of the chambers were wiped with wet swabs to remove migrated cells. The outer wall of the chambers were gently rinsed with PBS and stained with Giemsa (Sigma-Aldrich, St. Louis, MO) for 10 mins. Finally, the membrane was rinsed and allowed to air-dry. The membrane in triplicate was photographed and the number of cells counted.

### Invasion assay

Invasion assay was performed by a modification of the method described previously. A 100 μL Matrigel (Becton Dickinson, Franklin Lakes, NJ) was diluted to 1 mg/mL in serum-free RPMI. This solution was added to each upper chamber of the transwell with 8 μm pore size membrane. After solidification of Matrigel at 37 °C, approximately 1 × 10^5^ cells in serum-free RPMI were seeded onto the Matrigel over the upper chamber, followed by the addition of 250 μL of RPMI containing 10 % FBS at the bottom insert-well. After incubation of the cells at 37 °C for 18 h, the inner wall of the chambers were wiped with wet swabs to remove the cells, while the outer wall of the chambers were gently rinsed with PBS and stained with the Giemsa stain solution for 10 mins. Finally, the membrane was rinsed and allowed to air-dry. The membrane in triplicate was photographed and the number of cells counted.

### Western blot analysis

The electrophoretic experiments were performed by SDS-PAGE (10 % polyacrylamide unless specified otherwise) to analyze the YKL-40 and EMT related genes. Electrophoresis was conducted by a vertical gel electrophoresis device that was powered by (Mini PIII, Bio-Rad, Hercules, CA) a PAC 300 power supply (Bio-Rad). All SDS-PAGE samples (20 μg) were equilibrated in 10 mM Tris–HCl and 5 % SDS (pH 7.6) before loading.

Following complete separation, the gel was soaked briefly in a transfer buffer, which contained 25 mM Tris, 192 mM glycine, 20 % methanol, and 0.0375 % SDS (pH 8.3), for 30 s. The gel was then immediately electrotransfered to a nitrocellulose membrane (Hybond-ECL extra; Amersham) at 90 mA for 60 mins in a semi-dry Transfercell (Bio-Rad). The membrane was immersed in 2 % skim milk for 1 h with gentle agitation. After three washes with PBS for 5 mins each, the membrane was subjected to react with monoclonal or polyclonal antibodies and developed with chemiluminescence agents.

### Reverse transcription polymerase chain reaction (RT-PCR)

The YKL-40 mRNA levels in the NSCLC cells was determined by RT-PCR. Total RNA was extracted from the cells using TRIzol (Invitrogen Corporation, San Diego, CA). Following spectrometric determination of RNA yield, cDNA was synthesized with oligo (dT) primer using Moloney Murine Leukemia Virus (MMLV) Reverse Transcriptase. An aliquot of cDNA was subjected to 35 cycles of PCR using a standard procedure of initiation at 65 °C for 5 mins, incubating at 37 °C for 2 mins, and inactivating at 70 °C for 15 mins. The amplified products were resolved in a 1 % agarose gel and visualized by Sybr safe staining.

### Bioinformatics

The associations between gene expression levels and NSCLC patients prognosis were obtained from the PrognoScan database [16]. PrognoScan is a large collection of publicly available cancer microarray datasets with clinical annotation and a tool for assessing the biological relationship between gene expression and prognosis. In the PrognoScan database, association of gene expression with survival of patients was evaluated by the minimum P value approach. Briefly, patients were first arranged by expression levels of a given gene. They were then divided into high- and low-expression groups at all possible cutoff points, and the risk differences between any 2 groups were estimated by the log-rank test. Finally, the cutoff point that resulted the most pronounced P value was selected.

Meanwhile, the mRNA expression of YKL-40 among cancer patients and healthy population were mined from The Cancer Genome Atlas (TCGA) database and analysed using BoxPlotR.

### Statistical analysis

Data are presented as mean ± standard deviation (SD) The difference between the groups were calculated by Student t test (2 tailed). A *p* value of < 0.05 was taken as the indication of statistical significance.

## Results

### YKL-40 was highly expressed in NSCLC patients and was correlated with poor prognosis

**Fig. 1:**
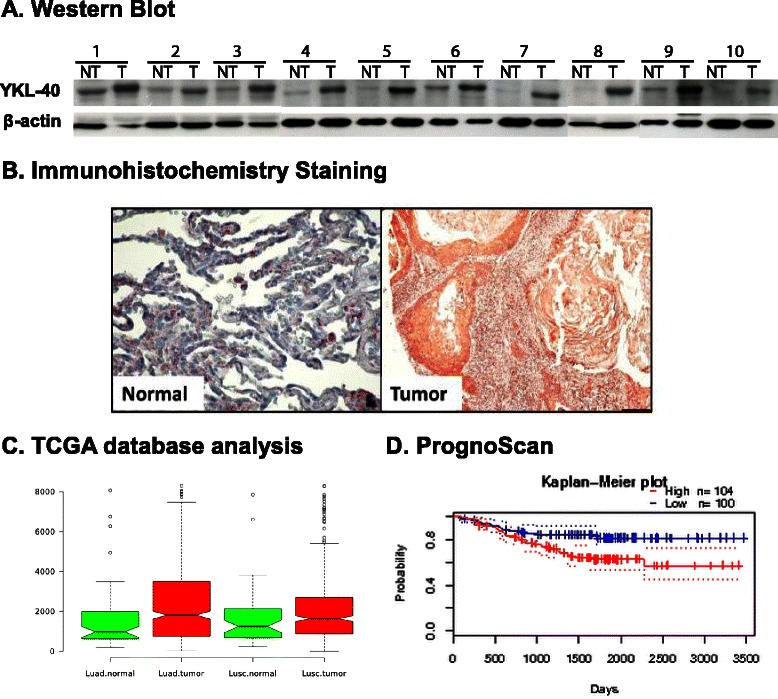
**a** Western Blot analysis comparison of YKL-40 gene expressions in normal lung tissues and non-small cell lung cancer (NSCLC). **b** YKL-40 immunohistochemistry staining comparison between normal lung tissue and lung tumor tissue (200 x magnification). **c** YKL-40 expression comparison analysis between normal lung cell and lung adenocarcinoma (Luad) & Lung squamous carcinoma (Lusc), *, *P* < 0.05, **, *P <* 0.005 (**d**) The correlation between YKL40 expression and NSCLC patient prognosis. Comparison of YKL40 expression and patient prognosis was examined using the Prognoscan database

Several NSCLC patients were investigated in this study, where consistently higher expressions of YKL-40 were observed in tumor than in non-tumor cells (Fig. 1a). Similar result were found in our immunohistochemistry staining analysis that compared the YKL-40 expression of normal lung cells and lung tumor cells (Fig. 1b). These results were further confirmed through TCGA analysis, by comparing YKL-40 expression between normal and lung adenocarcinoma as well as lung squamous carcinoma populations. The analysis determined that YKL-40 expressions were highly upregulated among lung carcinoma populations (Fig. 1c). Finally, we investigated the correlation of YKL-40 expression and NSCLC patient prognosis using the PrognoScan database (http://www.prognoscan.org/). As shown in Fig. 1d, high expression of YKL-40 was correlated with poor NSCLC patient prognosis.

### YKL-40 was correlated with tumor migration and invasion in NSCLC

**Fig. 2:**
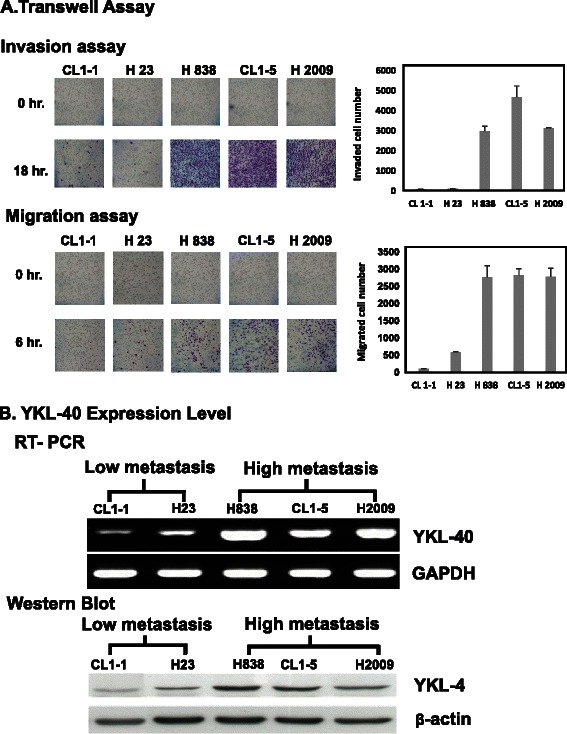
The effect of YKL-40 expression level on the invasion and migration abilities of the NSCLC cell lines. **a** The five NSCLC cell lines were divided into low metastasis (CL1-1, H23) and high metastasis (H838, CL1-5, H2009) groups by invasion and migration assay. **b** The YKL-40 expression level analysis by reverse transcription polymerase chain reaction (RT-PCR) and Western blot: indicating that the low and high metastasis groups expressed reduced and increased levels of YKL-40 mRNA and protein, respectively

To investigate the effect of YKL-40 expression level for tumor migration and invasion in NSCLC, five NSCLC cell lines (CL 1–1, CL 1–5, H23, H838, and H2009) were used for the evaluation of their migration/invasion abilities and YKL-40 expression levels. As shown in Fig. 2a, cell lines that had high migration activities were correlated, in general, with their high invasion. The cells were divided into low (CL 1–1 and H23) and high (H838, CL 1–5 and H2009) migration/invasion groups. Figure 2b shows that the cell lines with higher migration/invasion (H838, CL 1–5 and H2009) had greater YKL-40 mRNA and protein expression levels (determined by RT-PCR and Western blot), when compared with those that exhibited lower migration/invasion abilities (CL 1–1 and H23).

### Tumor invasion/migration effect of YKL-40 in NSCLC cell line

**Fig. 3:**
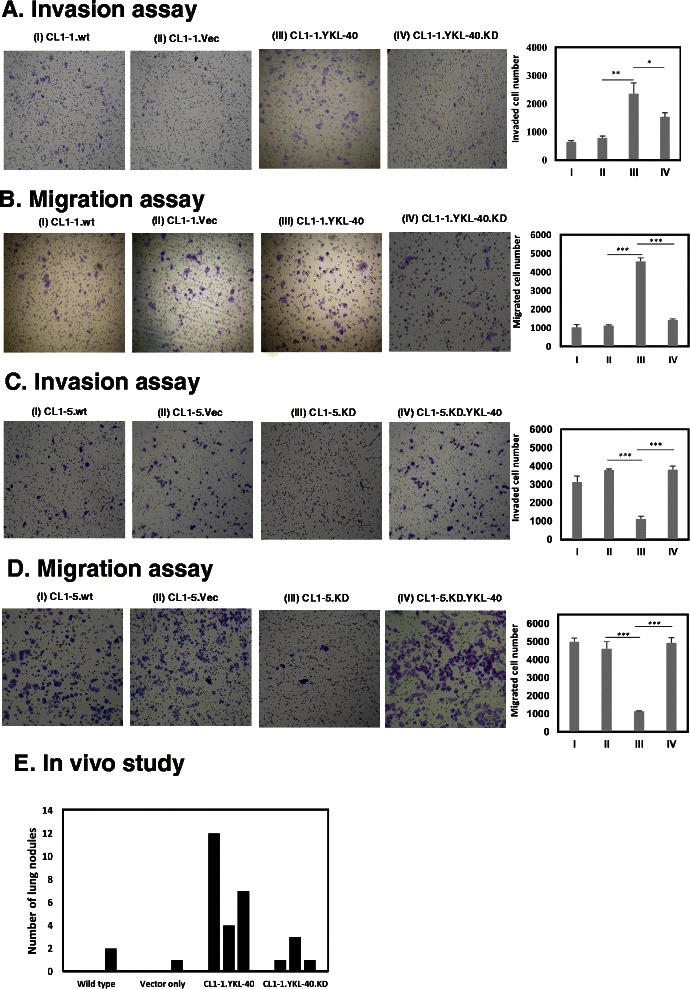
**a** and **b** CL1-1 cells (I) were transfected with the green fluorescent protein (GFP) expression plasmid (pEGFP-C1) plasmid, encoding empty vector (II) or full-length YKL-40 (III). After stable expression, cells were knockdowned via shRNA against human YKL-40 (IV). Four cells were subjected to Matrigel invasion assay (**a**) and transwell migration assay (**b**). **c** and **d** CL1-5 cells (I) were transfected with shRNA vector control (II) or shRNA against human YKL-40 (III). The cells were then co-transfected with the DsRed plasmid encoding YKL-40 (IV). The ability of these four cells to invade through Matrigel (**c**) or to migrate (**d**) was assayed. Mean ± transwell assay was determined by three independent experiments; statistical significance was measured using one way ANOVA, *, *P <* 0.05, **, *P <* 0.01, ***, *P <* 0.001. **e** in vivo study of each transfected cell lines’ migration ability. Each transfected cell lines were injected into nude mice for 6 weeks before lung harvest and lung nodules number determination. This experiment was conducted in triplicates for each group

**Fig. 4:**
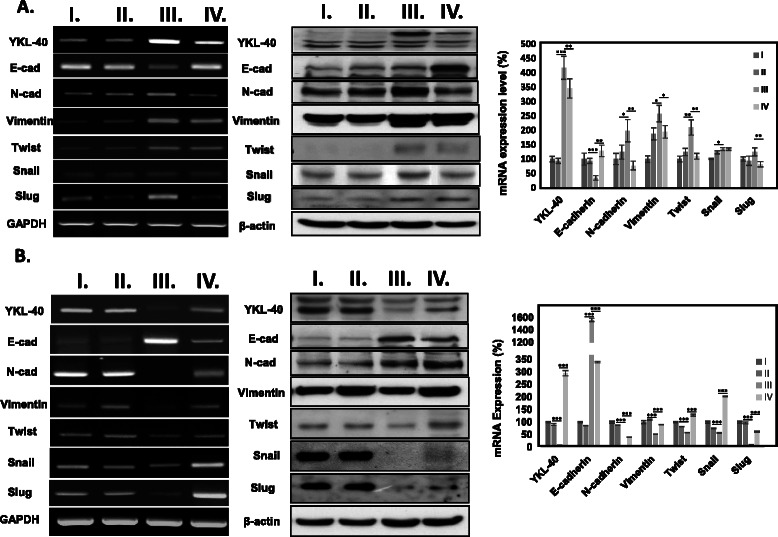
The epithelial–mesenchymal transition (EMT) marker expression regulation in modulated YKL-40 gene expression NSCLC. **a** EMT markers expression analysis of YKL-40 overexpressed CL1-1 cell (I) and YKL-40 reknockdown of YKL-40 overexpressed CL1-1 cell, as well as **b** EMT markers expression analysis of YKL-40 knockdowned CL1-5 cell (I) and YKL-40 re-overexpression of YKL-40 knockdowned CL1-1 cell using RT-PCR and Western Blot analysis. Mean ± transwell assay were determined by three independent experiments; statistical significance was measured using one way ANOVA, *, *P <* 0.05, **, *P <* 0.01, ***, *P <* 0.001

To investigate the effect of YKL-40 on cancer cell migration/invasion, YKL-40 knockdown in CL1-5 cells and YKL-40 overexpression in CL1-1 cells were constructed. After establishing the stable knockdown and overexpression cell lines, YKL-40 gene was transfected to re-overexpress in YKL-40 knockdown CL1-5 cells and re-knockdown of the CL1-1, YKL-40 overexpressed-cells. The YKL-40 expression by RT-PCR and Western blot in our cell lines are shown in Fig. 4a and b. The YKL-40 mRNA expression was substantially attenuated in YKL-40 knockdowned-cell and overexpression-reknockdown cell, when compared with cells without YKL-40 knockdown or the vector control. The YKL-40 gene was down-regulate to 6.9 % in the YKL-40 knockdowned CL 1–5 and had 35 %/22 % decrease in invasion/migration, when compared with the controls (Fig. 3a, b). The YKL-40 gene was up-regulated to 825 % in YKL-40 overexpression CL 1–1, which exhibited a 370 %/448 % increase of invasion/migration when compared with the controls (Fig. 3c, d). The YKL-40 overexpression-reknockdown CL1-1 cell was demonstrated to reverse its invasion/migration ability (Fig. 3a and b). Similarly, the YKL-40 knockdowned CL1-5 cell exhibited decreased ability of invasion/migration, when compared with the CL1-5 wild type. The invasion/migration ability of YKL-40 knockdowned CL1-5 cell was reversed when YKL-40 was re-overexpressed (Fig. 3c and d). Furthermore, the metastatic abilities of these cells were also confirmed in vivo. Four CL1-1 cell lines (WT, vector only, YKL-40 overexpression, and YKL-40 knockdown-reoverexpression) were injected into nude mice for 6 weeks and the number of metastatic lung tumor nodules were determined. The number of tumor nodules of the YKL-40 overexpression cells were significantly higher than those of the other groups (Fig. 3e). Overall, the results showed that an increase of YKL-40 expression can significantly enhance cancer migration/invasion and vice versa. This suggest that YKL-40 expression is associated with of NSCLC cells migration/invasion.

### YKL-40 promotes cell migration/invasion ability via regulating EMT related genes in NSCLC cells

To investigate the correlation between YKL-40 and EMT related-genes for promoting migration/invasion capability of NSCLC, several EMT-associated genes such as adhesion markers E-cadherin and N-cadherin; transcription regulators Twist, Snail and Slug; and cytoskeleton organization marker Vimentin in YKL-40 overexpressed-CL 1–1 and YKL-40 knockdowned-CL 1–1 (Fig. 4) were analyzed. The results suggested that mesenchymal markers (N-cadherin and Vimentin) were up-regulated and epithelial markers (E-cadherin) was down-regulated in YKL-40 overexpressed CL1-1. Moreover, EMT activators (Twist, Snail and Slug) were also up-regulated after YKL-40 overexpression in CL1-1. The results can also be reversed after YKL-40 gene reknockdown of YKL-40-overexpressed-CL1-1. Mesenchymal makers and regulators, and epithelial markers were determined to be decreased and increased, respectively, in YKL-40 overexpression-reknockdowned CL1-1, when compared to YKL-40 overexpressing CL1-1 (Fig. 4a). Likewise, YKL-40 knockdown CL1-5 exhibited repressed mesenchymal markers, and enhanced epithelial markers mRNA and protein level expression. However, the YKL-40 knockdown-reoverexpression CL1-5 cells underwent complete EMT markers expression reversion (Fig. 4b).

## Discussion

In the past decades, numerous of studies have found genes with modulated expressions in cancer cells. Most of these modulated genes hold important roles that involved sustained proliferative signaling, evading growth suppressors, activating invasion and metastasis, enabling replicative immortality, inducing angiogenesis, and resisting cell death [17]. Among all of these genes, multitude of clinical studies have determined that an abnormal up-regulation of YKL-40 is associated with cancer metastasis and poor patient survival [6, 18, 19]. In our study, each highly migrating/invading NSCLC cell lines (CL1-5, H838, and H2009) and surgical specimens from NSCLC patients exhibited higher expression of YKL-40 when compared to the controls (Figs. 1 and 2). Moreover, the expression of YKL-40 was determined to positively correlate with poor prognosis in NSCLC patients using the PrognoScan database (Fig. 2a). A number of studies have indicated that high serum levels of YKL-40 are correlated with higher metastasis and poor survival in a variety of human carcinomas [5, 17, 20]. At the time of diagnosis, 39 % to 91 % of patients with metastatic disease had elevated serum levels of YKL-40 [5]. However, the relationship between metastasis and YKL-40 expression level in tissues is not clear. Many studies have suggested that YKL-40 may have a role in cancer cell proliferation, survival, and invasiveness during the inflammatory process around the tumor, angiogenesis, and remodeling of the extracellular matrix [15]. Thus, transwell migration/invasion assay was performed to investigate the role of YKL-40 in cancer migration and invasion. Our results indicated that after YKL-40 gene overexpression, the migration and invasion ability of CL1-1 NSCLC cell lines were increased significantly, and can be reversed after further YKL-40 knockdown (Fig. 3a). On the other hand, YKL-40 gene knockdowns exhibited attenuated migration and invasion ability in CL1-5 NSCLC cell line, where the cells regains mobility after YKL-40 re-overexpression (Fig. 3b). In the past few years, it has been understood that cancer cells undergo EMT reprogramming to equip the cells with progression-associated capabilities for migration/invasion [21].

Numerous observations supported the idea that EMT is an important event in the progression, invasion, and metastasis of carcinomas, which may be a permanent feature in carcinomas that have a particularly dismal prognosis [22–24]. In our study, we explored the role of YKL-40 in EMT and determined that YKL-40 induces EMT progression (Fig. 4). The results indicated that the expression of E-cadherin, a marker of epithelial cells, was significantly lower; and the expression of N-cadherin, Vimentin, markers of mesenchymal cells, was significantly higher in YKL-40 overexpressed cell line, when compared to the control group. Other EMT inducers (or regulators), such as Snail, Slug, and Twist, have also been shown with remarkably higher expressions. It was clearly demonstrated in our findings that YKL-40 expression level corresponds greatly with that of the EMT genes. This result implies that YKL-40 promotes the migration and invasion of cancer cell by regulating EMT genes. Previous studies have also reported that YKL-40 may play a role in the regulation of phosphatidylinositol 3 kinase (PI3K)/AKT/mTOR pathway or Ras/Raf/MEK/ERK cascade, which is one of the best-studied signal transduction pathways connected with tumor survival, transformation, invasion, and metastasis [25, 26]. In the meantime, the activation of PI3K/AKT axis is emerging as a central feature of EMT, which is a well-known cancer migration and invasion enhancement pathway [21]. This study may bridge the two previous findings, where YKL-40 was determined to promote migration and invasion of cancer cells by regulating EMT essential genes via AKT signaling pathway. Our results indicated that YKL-40 has the potential to be a therapeutic target for cancer therapy. Furthermore, the study on the therapeutic potential of YKL-40 has been conducted by Faibish et al. by using YKL-40 targeting monoclonal antibody, where tumor angiogenesis and growth was effectively inhibited [27]. Thus, it can be concluded that YKL-40 possess an important role in tumor initiation and progression.

## Conclusions

**Fig. 5:**
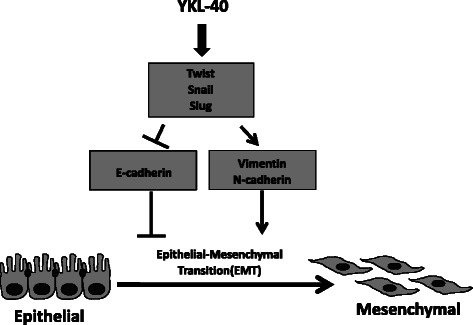
The schematic representation of EMT markers regulation by YKL-40, resulting in the induction of EMT

In conclusion, our finding revealed the important role of YKL-40 in promoting cancer metastasis. The expression level of YKL-40 corresponds highly with the migration and invasion ability of NSCLC. YKL-40 promotes cancer metastasis by regulating EMT essential genes (Fig. 5).

## Abbreviations

NSCLC: Non-small cell lung cancer
EMT: Epithelial-mesenchymal transition
TCGA: The Cancer Genome Atlas
WT: Wild-type
ANOVA: Analysis of variance
